# The UK consensus supporting effective introduction of novel treatments for multiple myeloma in the National Health Service

**DOI:** 10.1002/jha2.1038

**Published:** 2024-10-25

**Authors:** Rakesh Popat, Supratik Basu, Sarah Henshaw, Kamaraj Karunanithi, Karthik Ramasamy, Inderjit Singh, Anish Tailor, Ian Walker, Tim Warren, Noreen Ali, Charles Duffield, Gordon Cook

**Affiliations:** ^1^ Haematology University College London Hospitals NHS Foundation Trust London UK; ^2^ University of Wolverhampton The Royal Wolverhampton NHS Trust Wolverhampton UK; ^3^ Department of Clinical Haematology Nottingham University Hospitals NHS Trust Nottingham UK; ^4^ Clinical Haematology University Hospitals of North Midlands NHS Trust Stoke‐on‐Trent UK; ^5^ Oxford University Hospitals NHS Trust, Radcliffe Department of Medicine Oxford University Oxford UK; ^6^ Queen Elizabeth Hospital University Hospitals Birmingham NHS Foundation Trust Birmingham UK; ^7^ Triducive Partners Limited St Albans UK; ^8^ Pfizer Limited Tadworth UK; ^9^ Leeds Institute of Clinical Trial research & Leeds Cancer Centre Leeds Teaching Hospitals NHS Trust Leeds UK

**Keywords:** B‐cell maturation antigen, bispecific antibodies, Delphi study, multiple myeloma, United Kingdom

## Abstract

**Introduction:**

Multiple myeloma (MM) is a relapsing, debilitating blood cancer which remains incurable despite advances in treatments. Patients typically receive multiple lines of treatment, to which they become refractory, thereby limiting treatment options. B‐cell maturation antigen (BCMA) bispecific antibodies (BsAbs) represent a novel modality of treatment that has significant efficacy for relapsed or refractory patients.

The objective was to develop consensus statements for the effective implementation of BCMA BsAbs for relapsed or refractory MM patients within the National Health Service (NHS).

**Methods:**

The process employed a modified Delphi methodology. In March 2023, a literature review on the topic of novel treatments for MM was performed using the PubMed database.

The process employed a modified Delphi methodology. Following a literature review, a steering group of eight expert clinicians identified and agreed on five main topics of focus and 44 statements. These were then developed into an online survey which was distributed to healthcare professionals working in Levels 1, 2 and 3 haematology centres in the United Kingdom. Results were then shared with the expert panel to determine conclusions. The threshold for consensus agreement was set at 75%.

**Results:**

A total of 60 responses were received from all three centre levels. There was representation from all targeted centres. Consensus was achieved in 42 statements (95%) across three broad areas: the patient profile, initiation and step‐up dosing, monitoring and ongoing care, the role of multidisciplinary team and service designs for optimal management, consensus was not achieved for two statements. Given the level of agreement and that the stopping criteria were met, it was decided not to undertake further Delphi rounds.

**Conclusion:**

This consensus provides a framework to support the effective introduction of novel treatments for MM in the NHS. The results were used to inform a checklist for use within haematology services when considering the provision of MM care specific to BCMA BsAbs.

## 1 INTRODUCTION

Multiple myeloma (MM) is an incurable blood malignancy that develops in the bone marrow and involves the clonal expansion of abnormal plasma cells characterised by the secretion of monoclonal immunoglobulin [1, 2]. While MM is primarily a neoplastic disease of the bone marrow, the associated immunoparesis and secretion of abnormal immunoglobulin also contribute to multisystem sequelae. These include anaemia, bone lesions, hypercalcaemia and renal impairment/failure, which are components of the diagnostic “CRAB” criteria. Additional manifestations include recurrent infection, fatigue, hyperviscosity, amyloidosis and extramedullary disease [3].

MM accounts for 1%–1.8% of all cancer cases and is considered the second‐most common haematological neoplasm [1, 4]. Approximately 6000 patients are diagnosed every year in the United Kingdom, with around 3000 reported deaths. The highest incidence rates are reported in those aged 85–89, with more than 40% of cases diagnosed in those over 75 years old [4].

Over the last four decades, advancements in treatment have enhanced the survival of patients with MM, consequently, ten‐year survival has more than quadrupled with 29.1% of people surviving for 10 years or more compared with 6.4% in the 1970s [5]. With current treatments, survival is expected to now exceed this. However, patient survival is contingent on a combination of factors, including age, Revised International Staging System stage, comorbidities and cytogenetic risk [6].

Patients are typically exposed to the main treatment classes (proteasome inhibitors, immunomodulatory agents and anti‐CD38 monoclonal antibodies) early on in their treatment pathway and many can become refractory to these main classes. Real‐world data demonstrates that the outcomes of such triple‐class exposed patients are poor with an overall response rate of 29.8%, median progression‐free survival of 4.6 months and median overall survival of 12.4 months [7], signifying an unmet need. In addition, treatment‐refractory patients also experience deteriorated health‐related quality of life and elevated psychological distress [8, 9]. The prognosis for individuals with refractory disease remains poor, but the introduction of therapeutic agents with a different mechanism of action may improve this [10].

B‐cell maturation antigen (BCMA) is a member of the tumour necrosis factor receptor superfamily expressed by mature B‐cells, plasma cells and MM cells [11, 12]. BCMA has proved to be an important therapeutic target with different modalities of treatment being developed. Chimeric antigen receptor (CAR)‐T cells have also been developed to target BCMA with idecabtagene vicleucel and ciltacabtagene autoleucel now approved, but despite their efficacy, access to these treatments remains limited due to logistics, manufacturing constraints and cost [13].

Bispecific antibodies (BsAbs) are engineered to create an immune synapse between T‐cells and the target tumour cells. BCMA‐directed BsAbs bind BCMA on the MM cells and CD3 on T cells, triggering direct T‐cell activation and leading to the elimination of tumour cells [11, 12, 14]. Unlike CAR‐T cells, BsAbs are considered an off‐the‐shelf treatment which improves their accessibility to patients. Unlike CD38 monoclonal antibodies (mAbs), BsAbs have an adverse event profile that requires careful consideration. Due to their mechanism of action, BsAbs are associated with cytokine release syndrome (CRS) and immune effector cell‐associated neurotoxicity syndrome (ICANS) which require careful monitoring and management with patients remaining in close proximity to the hospital during initial step‐up dosing [11]. In addition, BCMA BsAbs are associated with an increased risk of infection and may need prophylaxis against infection, and intravenous immunoglobulin (IVIG) for hypogammaglobulinemia [15, 16].

Specific to the United Kingdom, teclistamab and elranatamab have been licensed in relapsed or refractory MM, demonstrating promising efficacy and safety results [17, 18], in addition, talquetamab, a BsAb targeting GPRC5D is also approved [19]. BsAbs have also been approved in lymphoma and acute lymphoblastic leukaemia demonstrating this treatment modality has activity across different disease types [20, 21, 22].

As an emerging technology, there has been limited experience with BCMA BsAbs and there is a need to agree on areas of service development to guide health care professionals. This is vital not only to ensure the delivery of a safe and efficient service but also to ensure there is equitable access across the country.

The aim of this project is to define the principles supporting the effective introduction of a new treatment for MM in the UK National Health Service (NHS) to achieve improved patient access and outcomes.

## 2 METHODS

**FIGURE 1:**
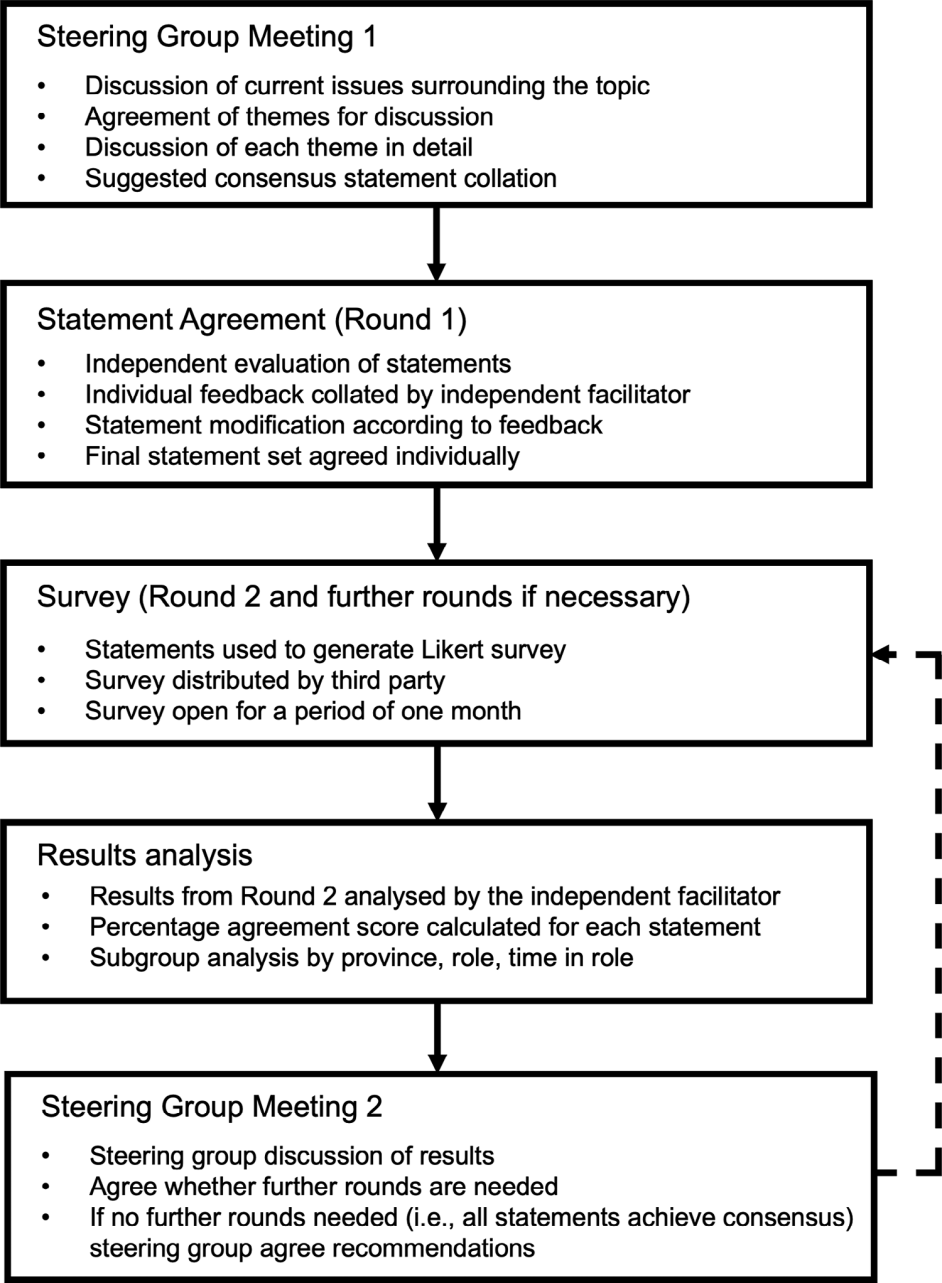
Modified Delphi study design.

The process followed a modified Delphi methodology (Figure 1). In March 2023, a literature review on the topic of novel treatments for MM was performed using the PubMed database. Search terms included but were not limited to: “incidence and prevalence”, “patient impact”, “societal and health costs”, “guideline recommendations” and “BCMA bispecific antibody therapies”. Guided by an independent facilitator, a steering group of UK specialists (five consultant haematologists/haemato‐oncologists, one Lead Myeloma Clinical Nurse Specialist, one Lead Haematology Pharmacist and one Chief Pharmacist) experienced in MM care convened in September 2023 to discuss the considerations for delivering BCMA BsAbs in the UK hospital setting. The steering group was selected based on published research, experience in MM service delivery and experience in delivering therapy for MM.

The information gathered from the literature review was used to inform the meeting discussion, during the discussion the steering group agreed on five main domains of focus:

1. Patient considerations
2. Initiation and managing step‐up dosing
3. Monitoring and ongoing care requirements
4. Multidisciplinary team (MDT) role
5. Service designs

These themes were discussed in detail and statements were suggested by the Steering Group. The statements were then collated, and the steering group independently rated the statements as either “accept”, “remove” or “reword” with suggested changes (as determined by a simple majority). This constituted the initial round of consensus.

The resulting 44 statements (Table 1) were developed into a Likert survey for distribution by a third party (M3) to a wider panel for round 2 of the process.

**TABLE 1:** Defined consensus statements and corresponding levels of agreement.

No.	Statement	Strongly Agree	Tend To Agree	Tend To Disagree	Strongly Disagree	Agreement
**Topic A: Patient profile considerations for bispecific therapies in myeloma**						
1	Existing frailty assessment tools should be incorporated into decision‐making when selecting appropriate patients who will have the ability to undertake and commit to treatment with a BCMA BsAb	50%	45%	2%	3%	**95%**
2	Patients outside of the above criteria should be considered on a case‐by‐case basis	57%	42%	2%	0%	**98%**
3	Patients with rapidly progressing disease may be more suitable for BCMA BsAbs over CAR‐T due to the potentially long preparation time for CAR‐T treatment	48%	38%	12%	2%	**87%**
4	Patients should be reviewed for comorbidities that may be exacerbated by BCMA BsAbs (such as active or recurrent infection) prior to starting treatment	57%	38%	3%	2%	**95%**
5	There is no evidence to support the use of bispecific agents with plasma cell leukaemia disease	22%	38%	37%	3%	**60%**
6	There is no evidence to support the use of bispecific agents with CNS disease	23%	38%	35%	3%	**62%**
7	Engaging with patients whose nine language is not English should involve interpreters/translated patient information	78%	18%	2%	2%	**97%**
8	As BsAbs may alter vaccine responses, patients being considered for treatment should have their vaccination status reviewed and optimised before commencing treatment	58%	35%	5%	2%	**93%**
**Topic B: Initiation and managing step‐up dosing**						
9	Admission protocols for step‐up dosing will be designed per the guidance in the SmPC	55%	40%	5%	0%	**95%**
10	Treatment should not be initiated outside of a supervised haematology ward with on‐site critical care	65%	25%	8%	2%	**90%**
11	As clinical experience with BCMA BsAbs grows, low‐risk patients may be able to undergo step‐up dosing in an ambulatory care setting, providing suitable protocols are in place for monitoring and escalation of care if required	40%	50%	8%	2%	**90%**
12	Hospital ward and on‐call medical, ambulatory care, nursing, and pharmacy teams should be familiar with CRS and ICANS treatment and management (including staging and grading)	70%	25%	5%	0%	**95%**
13	Hospital observation apps should include the means to assess CRS and ICANS scores using current assessment tools	58%	37%	3%	2%	**95%**
14	Tocilizumab vials should be available (with adequate supply) 24/7 in a known/specific location with on‐call pharmacy support wherever treatment is initiated	70%	27%	2%	2%	**97%**
15	Baseline cytomegalovirus (CMV) testing/recording (serology) should be undertaken prior to treatment initiation	60%	35%	3%	2%	**95%**
16	Patients who meet the criteria of the International Myeloma Working Group defined relapse should be promptly referred for consideration of treatment escalation	62%	33%	5%	0%	**95%**
17	High‐risk patients (those with high‐risk genetics, early relapse, extramedullary disease, etc.) should be discussed early, and before relapse, with the referral centre so an agreed patient pathway can be established	62%	35%	2%	2%	**97%**
18	Patients and their carers should be aware of the risk of CRS and ICANS prior to initiation of bispecific agents and know who to contact if they experience symptoms and have an appropriate alert card issued to identify treatment and risks	73%	20%	5%	2%	**93%**
19	Patients should be staged with a minimum of paraprotein and light chains; as well as whole‐body cross‐sectional imaging to assess for extramedullary disease	60%	32%	7%	2%	**92%**
20	Pharmacy risk assessment should be performed, and protocols established for the correct preparation of the BCMA BsAbs	62%	35%	3%	0%	**97%**
21	There is a need for patients to be initiated with an antimicrobial prophylaxis and antiviral treatment and antifungal treatment if they are considered high‐risk	65%	32%	2%	2%	**97%**
22	Access to the ICU should be available at any site initiating BCMA BsAb therapy and be alerted when required	83%	15%	0%	2%	**98%**
23	Inpatients receiving treatment with BCMA BsAbs should be discussed with day‐case colleagues to ensure continuity of care	57%	42%	0%	2%	**98%**
**Topic C: Monitoring and ongoing care requirements**						
24	CRS is likely to occur soon after initiation (within 1 week) and CRS with late doses is very uncommon	53%	40%	7%	0%	**93%**
25	Patients should be reviewed regarding their antimicrobial prophylaxis which may include antiviral treatment and antifungal treatment	60%	35%	5%	0%	**95%**
26	Regular IG monitoring should be performed at baseline and then 4‐weekly thereafter	55%	40%	5%	0%	**95%**
27	Patients with an IgG < 4 g/L should be considered for intravenous IG supplementation and given in line with national recommendations ("(i.e. Commissioning Criteria Policy for the use of therapeutic immunoglobulin (IgG) England, 2024, NHS)	43%	50%	7%	0%	**93%**
28	Subcutaneous IG could be an appropriate alternative to using Intravenous IG to reduce resource impact	50%	37%	12%	2%	**87%**
29	The frequency of intravenous IG replacement should be monitored and adjusted according to the frequency of infections and IG level	53%	45%	0%	2%	**98%**
30	Support for commissioning applications for intravenous IG would be helpful to aid in access	62%	33%	5%	0%	**95%**
**Topic D: MDT role**						
31	All decisions relating to patient selection for BCMA BsAbs should be agreed upon at a local MDT	67%	22%	10%	2%	**88%**
32	Effective delivery of MDTs improves referral times for patients	65%	30%	3%	2%	**95%**
33	MDTs need to be aware of the funding route (Blueteq criteria) for treatment options under consideration	58%	37%	3%	2%	**95%**
34	MDTs should be managed in line with improving outcomes guidance (Improving outcomes: a strategy for cancer (DOH 2011))	68%	28%	2%	2%	**97%**
35	There is a lack of clinical pharmacy resources to support MDTs	45%	30%	20%	5%	**75%**
36	MDTs should ideally include an infectious disease specialist when assessing a patient for treatment with BCMA BsAbs	40%	38%	20%	2%	**78%**
**Topic E: Service designs**						
37	Access to bispecific treatment should be available in all Level 2 (and above) haematology centres in the NHS	57%	32%	10%	2%	**88%**
38	Patients should be provided priority access to direct clinical nurse specialist colleagues and the oncology ward when needed	53%	42%	3%	2%	**95%**
39	Inpatient, daycare and ambulatory care services should be utilised to manage the capacity to deliver bispecific therapy	52%	45%	3%	0%	**97%**
40	Where the SmPC requires an aseptic technique, BCMA BsAbs products can be prepared outside of an aseptic unit, following an appropriate risk assessment	33%	42%	22%	3%	**75%**
41	Level 2 centres that cannot initiate these medicines should refer to (Hub and Spoke) and then manage ongoing care	48%	45%	7%	0%	**93%**
42	Level 1 centres could manage ongoing patients according to the hub and spoke model	40%	45%	15%	0%	**85%**
43	Hub centres should retain patients for the first cycle of treatment before returning care responsibility to the original spoke centre	50%	38%	10%	2%	**88%**
44	Turnaround time for virology should be 1‐week maximum	48%	43%	7%	2%	**92%**

Recruitment of panel members was according to the following criteria:

Employed within the NHS in the UK as:

1. Consultant Haematologist/Haemato‐oncologist (*n* = 40),
2. Haematology Nurse Specialist (*n* = 10) and
3. Pharmacist (*n* = 10)

The identity of the respondents was not known to the steering group or the independent facilitator. The survey presented each statement along with a 4‐point Likert scale (‘strongly disagree’, ‘tend to disagree’, ‘tend to agree’ and ‘strongly agree’) to allow respondents to indicate their corresponding level of agreement. The survey also captured some demographic data for further analysis. Demographic data captured included haematology centre level (1, 2 or 3), Country and professional role. All responses collected were included in the final analysis.

Stopping criteria were established a priori as a 2‐month window to collect responses (October 2023), a target of 60 responses, 90% of statements passing the threshold for consensus and a threshold for consensus set at 75% (a widely accepted threshold [23]). Consensus was then further defined to be ‘strong’ at ≥ 75% and ‘very strong’ at ≥ 90%. These criteria were established to gain the required number of responses while accounting for time pressures within the healthcare system.

A statement of consent was included at the start of the survey and consent was implied by completion. As this study only collected the anonymous opinions of healthcare professionals and no patient‐specific data was captured, ethical approval was not sought.

Completed surveys were analysed to produce an arithmetic agreement score for each statement using Microsoft Excel software. The responses were aggregated to provide an overall agreement level (i.e. the number of responses expressing agreement as a percentage of the overall number of responses for each statement). This information was then reviewed by the members of the steering group to determine what recommendations and conclusions could be developed based on the responses received.

## 3 RESULTS

Questionnaires were completed and received from a total of 60 respondents (Table 1). A subsequent analysis was conducted on the responses. All professionals were specialists actively engaged in the management of patients diagnosed with MM: Consultant Haematologist, *n* = 40; Nurse Specialist, *n* = 10; Pharmacist, *n* = 10 (Figure S1). Most respondents (*n* = 54) were based in England. Additionally, three in Wales and three in Scotland (Figure S2). Seven respondents from this study were based at a Level 1 haematology centre, 27 at a Level 2 centre and 26 at a Level 3 centre (see Appendix for definitions) as shown in Figure S3.

As the stopping criteria were satisfied, no additional testing rounds were conducted.

**FIGURE 2:**
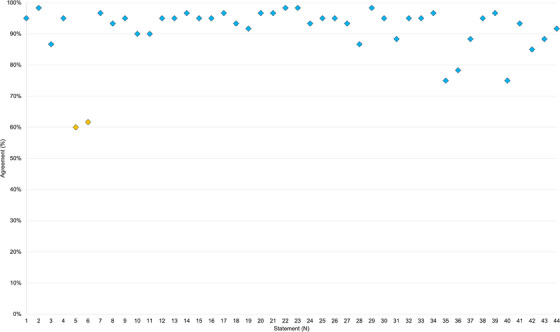
Consensus agreement levels by statement. The threshold for consensus is depicted by the green line (75%). The blue line signifies the threshold for very strong agreement (90%).

Consensus was achieved in 42 statements (95%), 33 of which with agreement levels above >90%. Consensus was not achieved (< 75%) in 2/44 (4.5%) statements (Figure 2).

The distribution of consensus scores on the 4‐point Likert scale is represented in Figure S4.

### 3.1 Topic A: patient profile considerations for BCMA BsAb therapies in myeloma

Responses to statements in Topic A emphasised the importance of individual patient assessment and patient profile consideration before initiating treatment. Consensus was established regarding the consideration of frailty assessment (S1, 95%), rate of progression (S3, 87%), assessment of comorbidities (S4, 95%) and vaccination status (S8, 93%) when selecting and preparing patients for treatment with BCMA BsAb therapies. A consensus was not achieved regarding the evidence base to support BsAb therapies in plasma cell leukaemia disease (S5, 60%), or central nervous system (CNS) disease (S6, 62%).

Respondents agreed on the importance of individual patient assessment and patient profile consideration before initiating treatment. Factors such as disease course, age, performance status, comorbidities and cytogenetic risk should be considered [24, 25]. Another factor that may influence the choice between CAR‐T and BsAbs is the product manufacturing times associated with CAR‐T, as BCMA BsAbs are an ‘off the shelf’ product, BCMA BsAbs could be a useful treatment option in patients with rapidly progressing disease [26].

### 3.2 Topic B: initiation and managing step‐up dosing

Very strong agreement (≥90%) was observed for all statements in this section. Considering the potential adverse events of BCMA BsAb therapy, implementing step‐up dosing of BCMA BsAbs requires vigilant monitoring and close communication with the patient. Moreover, respondents considered that standardised protocols for the risk assessment and administration of BCMA BsAbs should be in place.

### 3.3 Topic C: monitoring and ongoing care requirements

Respondents agreed (93%) that patients with immunoglobulin gamma (IgG) levels < 4 g/L should receive immunoglobulin support in line with the International Myeloma Working Group guidelines [27]. This contrasts with the NHS England Clinical Priorities Advisory Group (CPAG) and NHS Scotland guidance (see Appendix for criteria). This lack of concordance between clinician recommendations and IVIG commissioning criteria means that further work is required to achieve alignment. In addition, there was a slightly lower agreement that subcutaneous (SC) IG is an appropriate alternative to intravenous IVIG (87%). This difference in opinion may be attributed to individual preferences or centre experience; however, the steering group suggest that SCIG should be considered equivalent to IVIG, and the choice of product used should be locally agreed upon.

### 3.4 Topic D: MDT role (role of myeloma team vs. local, regional and national MDT)

Consensus, (≥75%) was achieved on all statements related to this topic. It is recommended that local MDTs should be in place to manage BCMA BsAb decisions and that these should adhere to the principles outlined in “Improving outcomes guidance: a strategy for cancer” [28]. Therefore a national MDT to agree on which BCMA‐targeted treatments should be offered to patients was not recommended as the consensus was that local meetings should be permitted to make these decisions.

### 3.5 Topic E: service designs (Hub and spoke, shared care and role of ITU vs. neurology vs. CAR‐T)

There was strong consensus (≥75%) across statements on this topic, supporting the preparation of BCMA BsAbs products outside of the aseptic unit (e.g. on the ward) providing appropriate systems are in place (including risk assessment) to ensure aseptic conditions, and delivery by suitably trained and accountable personnel (S40, 75%).

**FIGURE 3:**
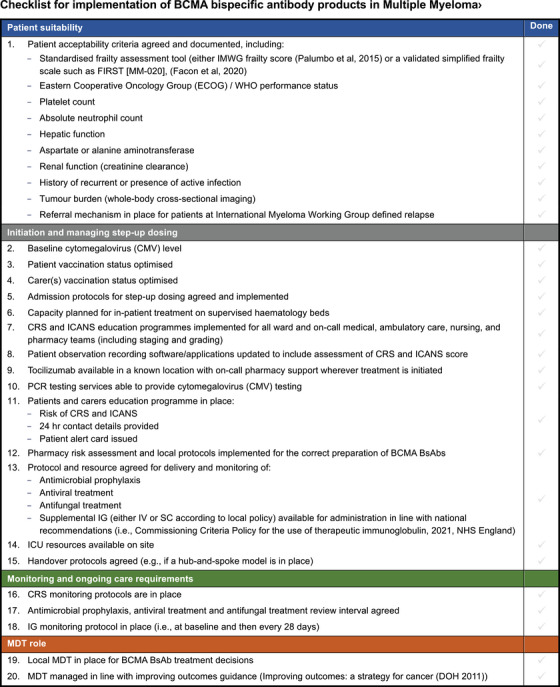
Checklist to support the implementation of B‐cell maturation antigen (BCMA) bispecific antibodies in multiple myeloma.

Based on the levels of consensus achieved, the authors propose the following recommendations and corresponding haematology services to use when considering the provision of MM care specific to BCMA BsAbs (Figure 3).

1. All Level 2 and above haematology centres should have access to BCMA BsAbs with hub and spoke models (a model comprising a highly expert centre (the hub) and a series of secondary units (the spokes) used to support use in less experienced centres)
2. All initiating centres require supervised haematology/ oncology beds with access to ICU/critical care
3. All centres require immediate access to tocilizumab with on‐call pharmacy support as required
4. Existing frailty assessment tools should be incorporated into decision‐making when selecting appropriate patients for treatment with a BCMA BsAb
5. Those who do not meet pre‐specified criteria for treatment should be considered on a case‐by‐case basis at an MDT
6. Individuals with rapidly progressing diseases who may not be suitable for CAR‐T treatment due to product preparation times may be suitable candidates for a BCMA BsAb
7. All hospital ward and on‐call medical, ambulatory care, nursing and pharmacy teams should be familiar with CRS and ICANS treatment and management (including staging and grading)
8. Patient pathways should allow for high‐risk/rapidly progressing patients to be referred early to BsAb centres to mitigate any system delays
9. Patients should be reviewed regarding their antimicrobial prophylaxis which may include antiviral treatment and antifungal treatment
10. Protocols should be in place for the standardised nurse‐led preparation of BCMA BsAb products on the ward where feasible

## 4 DISCUSSION

Multiple myeloma remains an incurable disease, and over time it is likely that the majority of sufferers will become refractory to multiple lines of treatment, leaving few treatment options available. The introduction of BCMA BsAbs provides a valuable treatment option for these patients, but as a new treatment modality, there is a need to establish principles of use.

The consensus established in this study provides the basis for recommendations regarding the effective introduction of BCMA BsAbs in MM, the only areas where consensus was not established were on the existence of evidence to support the use of BsAbs in plasma cell leukaemia or CNS‐involved disease. This is likely to be due to a lack of published RCT evidence, as these are ongoing, but in the interim real‐world data for BsAbs is emerging. This data typically involves patients who would not meet the inclusion criteria for a clinical trial [29, 30, 31, 32]. It is probable that some respondents were aware of this evidence.

The steering group discussed the topic of monitoring for viral reactivation by blood polymerase chain reaction and underlined that not all hospitals may have the capability for cytomegalovirus testing within the required turnaround times.

Whilst respondents across all haematological level centres agreed on the potential to administer BCMA BsAbs in an outpatient/ambulatory setting, there would need to be a risk assessment performed to identify those at high risk of complications. The potential for severe side effects of BCMA BsAb treatment (including high‐grade CRS and ICANS) underlines the importance of patient and caregiver education to recognise and report any concerns for rapid clinical assessment (S18, 93%) to minimise these risks.

Emerging real‐world evidence suggests that patients with MM experience up to 4‐fold increased risk of infection compared to matched controls [33]. It is known that immune dysregulation is a distinctive feature of clinically active MM, and this is associated with an increased risk of infections and reduced responsiveness to vaccinations, necessitating the monitoring of post‐vaccination antibody titres or the need for repeated vaccinations [34, 35]. Additionally, immunosuppressive therapy also increases the risk of infections, underlying the importance of both the patient and any caregivers having their vaccination status assessed and optimised [35]. The risk of infection associated with BCMA BsAbs requires a high degree of vigilance and robust prophylaxis strategies [36, 37].

MDT definition and practice may vary regionally, and there is a resulting uncertainty regarding the availability of pharmacist resources to support the MDT as this could either be interpreted as “there is a lack of clinical pharmacy resources to support haematology MDTs”, or “there is a lack of clinical pharmacy representation in MDT meetings”. The steering group suggest, that in their experience clinical pharmacy resource is broadly available and supports daily MDT activity but may not formally attend MDT meetings. There may be value in future work to identify whether this assumption is correct and whether there is an opportunity for clinical pharmacy inclusion in formal MDT meetings.

Preparation of BCMA BsAbs products outside of the aseptic unit would reduce the burden on aseptic units and could be managed in a similar way to the preparation of monoclonal antibody treatments in some centres. This practice is not, however, universal and a standardised approach should be agreed upon and supported by a national guideline which also includes dose‐rounding/dose‐banding tables for specific BCMA BsAb products to support practice. As BCMA BsAb products may have differing requirements (e.g. some may require weight‐based dosing), local risk assessment and product preparation protocols should be in place for each BCMA BsAb treatment.

### 4.1 Strength and limitations

This consensus project achieved strong agreement from 60 experts working in MM in the UK, with responses from Level 1, 2 and 3 haematology centres to provide insight across different settings. The project was designed to capture not only the opinions of haematologists/haemato‐oncologists but also haematology nurse specialists, and pharmacists. The clearly defined inclusion criteria ensured that only the views of relevant stakeholders were sought. The use of a third party to manage the anonymous questionnaire distribution and subsequent collection of results reduces any bias from the steering group.

There are some key limitations identified by the authors, the first of which is the high levels of agreement achieved over one round of wider testing, this suggests potential bias in that the statements were designed to be agreeable and did not sufficiently challenge the status quo. Further research on this question should refine the statements generated herein to determine any greater variance that may exist. In addition, there is a significant bias towards practice and opinion in England (54/60 responses), suggesting that the recommendations may not transfer directly to practice in other devolved nations in the UK.

## 5 CONCLUSION

This modified Delphi exercise was able to achieve agreement from a panel of 60 healthcare professionals currently involved in the delivery of MM care across the UK for all but two statements. Health care professionals recognized the discrete challenges but were keen for the successful implementation of BCMA BsAbs as a treatment option for MM patients within the NHS to provide a safe and effective service.

## AUTHOR CONTRIBUTIONS

Rakesh Popat acted as the corresponding author for the submission process, Rakesh Popat, S. Basu, S. Henshaw, K. Karunanithi, K. Ramasamy, I. Singh, and A. Tailor acted as the Steering Group for this study, developed and reviewed initial statements, contributed to the analysis and discussion of results equally and reviewed, edited and approved the final manuscript. I. Walker, and T. Warren acted as facilitators for the Steering Group meetings, managed the modified Delphi process and drafted survey materials and the initial draft manuscript. C. Duffield and N. Ali conceptualisation, writing support and project management.

## CONFLICT OF INTEREST STATEMENT

**RP**, **SB**, **SH**, **KK**, **KR**, **IS** and **AT** received honoraria from Pfizer while undertaking this study. Pfizer commissioned Triducive Partners Limited (**IW** and **TW**) to facilitate the project and analyse the responses to the consensus statements in line with the Delphi methodology.

## ETHICS STATEMENT

The authors have confirmed ethical approval statement is not needed for this submission.

## PATIENT CONSENT STATEMENT

This was not required as the stated objective was to examine the opinions of healthcare professionals towards the utility of BCMA BsAbs in MM.

## CLINICAL TRIAL REGISTRATION

The authors have confirmed clinical trial registration is not needed for this submission.

## Supporting information




**FIGURE S1**: Occupational distribution of respondents.

**FIGURE S2**: Distribution of respondents in the UK by country.

**FIGURE S3**: Distribution of respondents based on the Haematology centre level.

**FIGURE S4**: Distribution of responses across agreement levels by statement.

## Data Availability

The datasets used and/or analysed during the current study are available from the corresponding author upon reasonable request.

## Haematology centre levels

According to the British Committee for Standardisation in Haematology (BCSH), levels of care are broadly defined as one of three categories according to the complexity of the treatment delivered; the duration of anticipated neutropenia following chemotherapy; and the rarity of the disease subtype. (levels 1, 2 and 3). Broadly, Level 1 centres offer treatments that are not expected to result in significant bone marrow suppression and neutropenia, Level 2 offers treatments associated with significant neutropenia and inpatient care and Level 3 the most complex in‐patient care including for rare diseases [38].

## The NHS England Clinical Priorities Advisory Group & NHS Scotland guidelines criteria for immunoglobulin support

- Recurrent or severe bacterial infection despite continuous oral prophylactic antibiotic therapy for 6 months [39, 40];
- Serum IgG < 4 g/L (excluding paraprotein);
- Documented failure of serum antibody response to unconjugated pneumococcal or other polysaccharide vaccine challenge (note: vaccine challenge may be of limited value in patients with very low serum IgG (< 3 g/L)).

*Note: NHS England acknowledges that not all criteria (a)–(c) will need to be fulfilled for an individual patient* [40].
