# Supplementary figures and images for: The UK consensus supporting effective introduction of novel treatments for multiple myeloma in the National Health Service
Source: EJHaem. 2024 Oct 25;5(6):1133–43. doi: 10.1002/jha2.1038 (PMC11647736; doi:10.1002/jha2.1038)

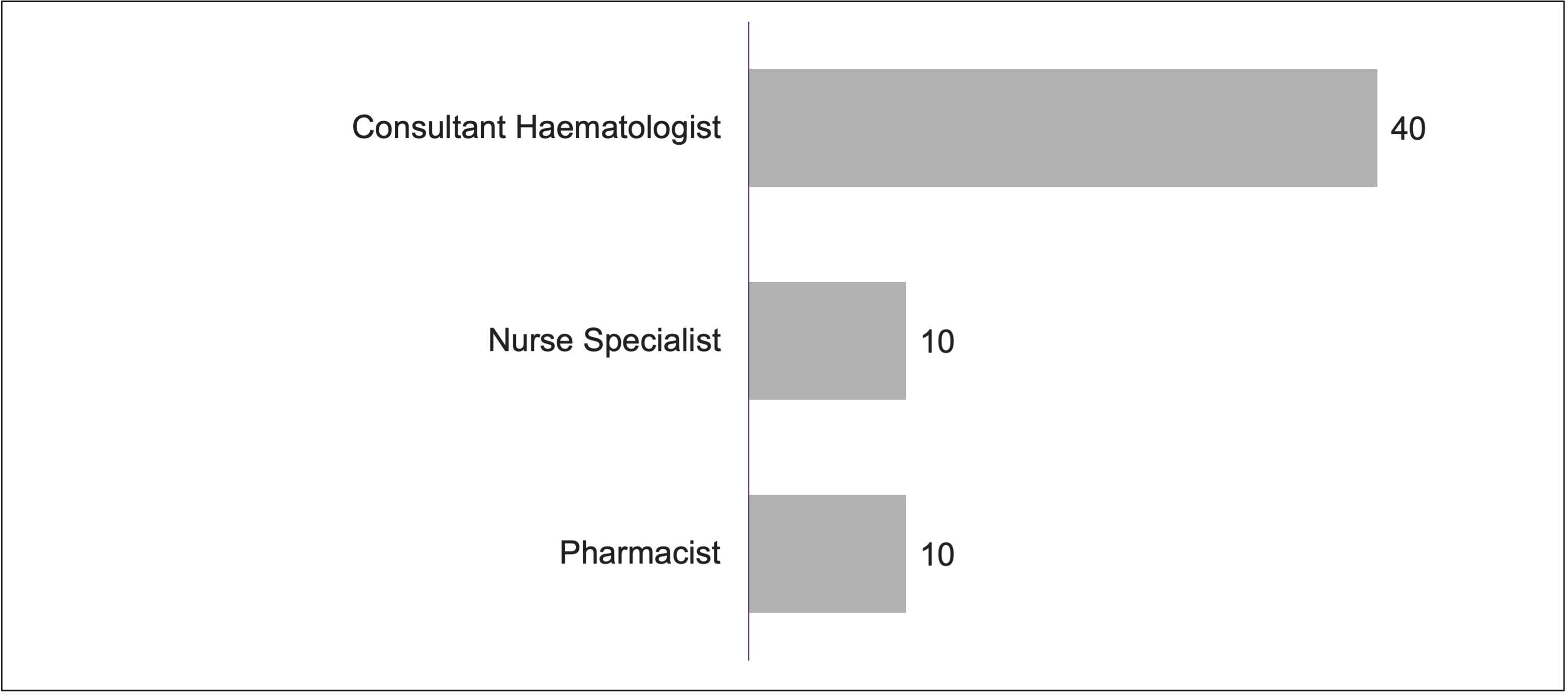

Supplement: Supplementary file 1 — FIGURE S1: Occupational distribution of respondents. [file JHA2-5-1133-s002.png]

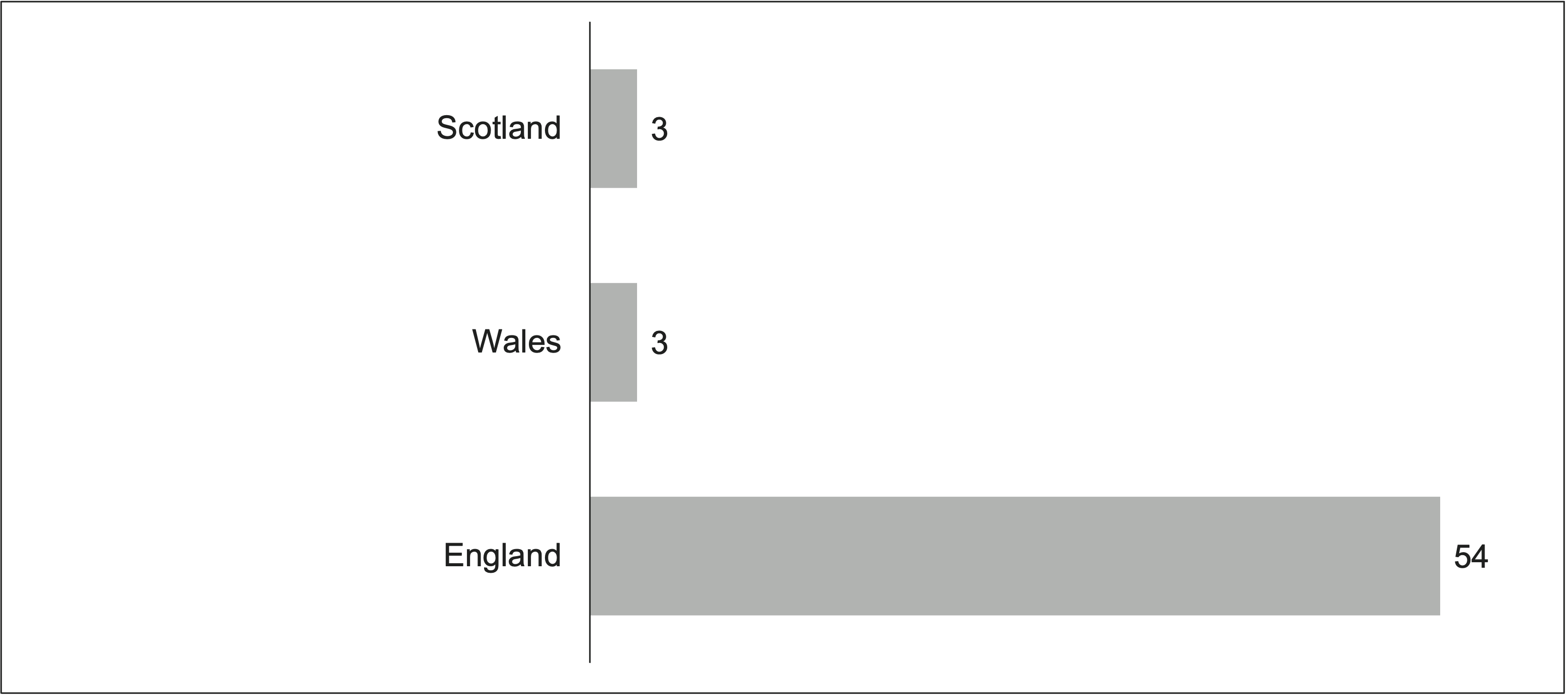

Supplement: Supplementary file 2 — FIGURE S2: Distribution of respondents in the UK by country. [file JHA2-5-1133-s004.png]

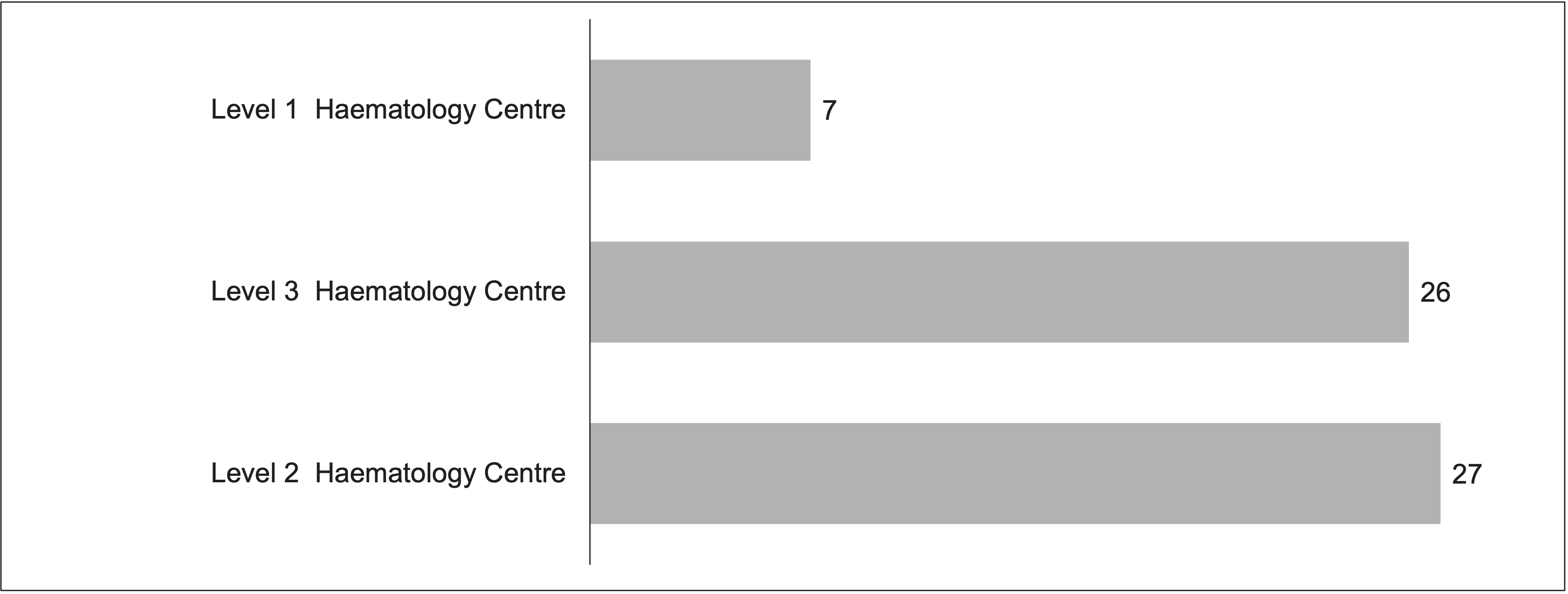

Supplement: Supplementary file 3 — FIGURE S3: Distribution of respondents based on the Haematology centre level. [file JHA2-5-1133-s003.png]

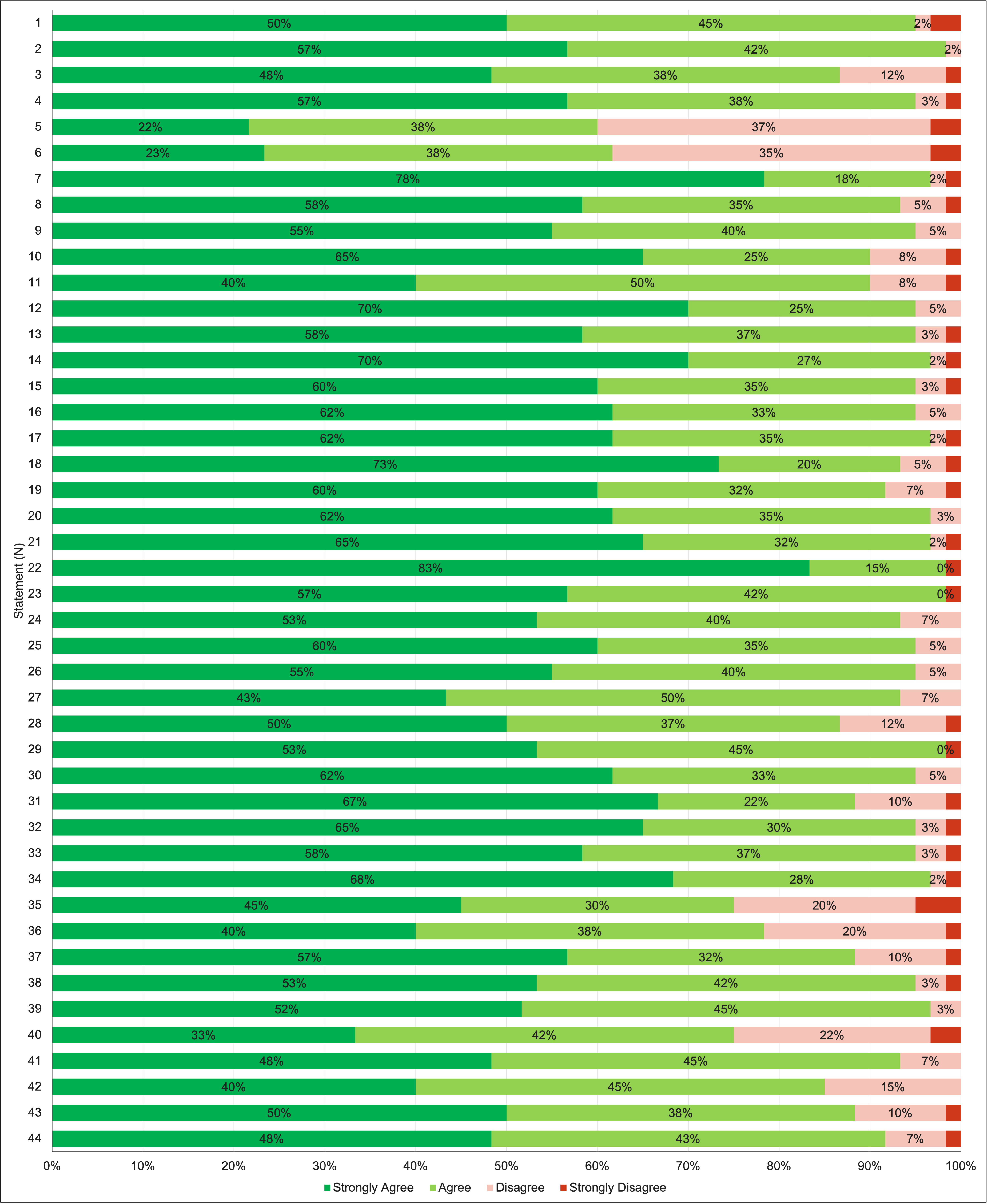

Supplement: Supplementary file 4 — FIGURE S4: Distribution of responses across agreement levels by statement. [file JHA2-5-1133-s001.png]
